# Pennsylvania Hospital

**Published:** 1842-01-08

**Authors:** 


### CLINICAL REPORTS.

Pennsylvania Hospital—*Surgical Wards—Service of* Dr. E. Peace.

*Acetate of Lead as a counter-agent to Fetor in sloughing, and other sores.*—The two following notes are chiefly interesting as instances of the apparently good effects of the lead-water dressing on some sloughing surfaces. Reference has been made already to this application of the remedy, in a previous report,* and will be considered more at large in a future communication.

E. H.

1. A recent case of caries of the malar bone in a child six years of age, was treated merely with emollient applications for four months.

* See Medical Examiner, Vol. IV. page 776, 1841.

During this time, two pieces of bone—the first of them being a portion of the orbitar process, and nearly half an inch long and a quarter of an inch wide—came away through an opening just outside of, and below the external angle of the right eye, leaving a cavity, from which constantly issued a very fetid discharge. At the end of four months, the surface of the bone, being covered with granulations, could no longer be felt with the probe. The part was then dressed with lint kept moistened with diluted *liq. sodæ chlorid.*, to correct the offensive odour of the discharge which still persisted. This had the desired effect to a considerable extent, but did not entirely remove the evil. A weak solution of the acetate of lead was at length directed to be kept continually applied with a piece of lint over and around the orifice, while a small portion of the same was injected every day into the little cavity still remaining in the cheek. The lead water completely destroyed the fetor; the cavity began to fill up rapidly, and in about ten days it was entirely closed.

2. In a severe case of onychia after small pox in a little girl aged six years, the nail was removed and the finger covered with emollient fomentations. A fermenting poultice was substituted in a few days, on account of sloughing. This abated immediately, and for a short time disappeared under the appplication. Although the finger two or three times appeared to be recovering under the use of nitrate of silver and other escharotic and astringent applications, yet it as often relapsed without any obvious local cause, and resumed its offensive, sloughy aspect. At length, resort was had to the acetate of lead, a solution of which was kept steadily applied to the affected part by means of patent lint covered with oiled silk. Under this dressing alone, the dark slough disappeared, the fetor became imperceptible, and the little patient soon entirely recovered, with the loss of the nail.

